# Genome-wide identification and analysis of the HD-Zip transcription factor family in oats

**DOI:** 10.3389/fmolb.2024.1475276

**Published:** 2024-10-30

**Authors:** Yiqun Xu, Changlai Liu

**Affiliations:** ^1^ Co-Innovation Center for Sustainable Forestry in Southern China, Bamboo Research Institute, Nanjing Forestry University, Nanjing, China; ^2^ College of Agriculture, Shanxi Agricultural University, Jinzhong, Shanxi, China

**Keywords:** HD-ZIP gene family, oats (*Avena sativa*), abiotic stress, expression analysis, transcriptome analysis

## Abstract

**Introduction:**

HD-Zip transcription factors are an important class of plant transcription factors involved in regulating plant growth and development as well as various stress responses. In order to explore the characteristics of oat HD-Zip transcription factors and their transcriptomic expression patterns under abiotic stress, this study identified members of the HD-Zip gene family in the oat genome through bioinformatics methods and analyzed their basic physicochemical properties, evolutionary relationships, conserved structural domains, gene duplication relationships, and expression profiles.

**Results:**

74 HD-Zip gene sequences were identified in the oat genome, unevenly distributed in all chromosomes except the 4D chromosome. The 74 HD-Zip genes can be divided into four subfamilies (HD-Zip Ⅰ-Ⅳ), containing 30 (HD-Zip Ⅰ), 38 (HD-Zip Ⅱ), 4 (HD-Zip III), and 2 (HD-Zip IV) genes, respectively. The grouping of this study is completely consistent with the clustering results of family members in the oat HD-Zip gene phylogenetic tree, further supporting the reliability of the sequence grouping. In addition, there are significant differences in conserved motifs and gene lengths between subfamilies, but they are conserved within the same subfamily. Under drought and salt stress, the expression levels of a large number of oat HD-Zip genes were significantly induced or suppressed, indicating that these HD-Zip genes are likely to be involved in regulating the oat’s response to adversity.

**Conclusions:**

The results of this study provide excellent candidate HD-Zip genes for the study of oat resistance, and provide a reference for oat gene improvement and genetic breeding.

## 1 Introduction

Oats (*Avena sativa* L.) are an annual herbaceous plant of the oat genus (*Avena*) in the Poaceae family, and are an important economic crop of the Poaceae family. The three subgenomes of cultivated oats are derived from two ancestral species, *A. longiglumis* (2n = 2x = 14, AlAl genome) and *A. insularis* (2n = 4x = 28, CCDD genome), through interspecies hybridization, forming the current allohexaploid form (AACCDD, 2n = 6x = 42) (Peng et al., 2022). Oats can be grown alone or intercropped with annual legumes to provide hay and green fodder. In the cold temperate regions of the Northern Hemisphere, it is an important crop in Canada, Russia, and Nordic countries (He and Åsmund, 2012), and is mainly grown in the northern part of Shanxi in China. Oats, with their good seeds, high yield, and good quality, have always played a prominent role in China’s livestock production. In addition, compared with rice, wheat, and other forage crops, oats have strong resistance (Canales et al., 2021) and can be used as a pioneer crop for soil improvement (Han et al., 2014). Therefore, the excavation of oat stress-resistant transcription factor genes is of great significance for the genetic improvement of oats and the promotion of stress-resistant breeding of staple food crops (i.e., using the drought-resistant genes of oats to improve staple food crops).

Transcription factors (TF) refer to proteins that bind to DNA and interact with specific regions in the promoter region of eukaryotes (Singh et al., 2002). The HD-Zip (Homeodomain-leucine zipper protein) gene family is a class of transcription factors unique to higher plants, playing an important role in plant growth, development, environmental adaptation, and stress responses. The HD-Zip transcription factor family is a group of transcription factors unique to higher plants. Increasing research has shown that HD-Zip transcription factors are important regulators in plant responses to biotic and abiotic stresses (Deng, 2014). The *Hahb-4* gene found in sunflower significantly increases drought tolerance when overexpressed in Arabidopsis (Manavella et al., 2006). Ariel and colleagues isolated a *MtHB1* gene from alfalfa, and functional analysis showed that this gene can enhance plant tolerance to salt stress (Ariel et al., 2010). All members of its family contain a homeodomain (HD) composed of 60 amino acids and an adjacent leucine zipper-loop-zipper motif (LZ) (Johannesson et al., 2001). The HD domain is encoded by a homeobox (HB) sequence of 180 bp (or 183 bp) in length, which is responsible for binding to specific DNA sequences (Dicristina et al., 1996), while the leucine zipper conserved motif (LZ) is responsible for promoting protein-protein interactions (Wang et al., 2013). And according to the sequence conservation, protein function, gene structure and other factors of the protein, the HD-Zip family can be divided into four subfamilies: HD-Zip Ⅰ-Ⅳ (Sessa et al., 2018). Members of the HD-Zip Ⅰ gene family play an important role in plant stress response, organ development, light signal response, and hormone response (Johannesson et al., 2003); members of the HD-Zip II subfamily are mainly related to auxin response (Arce et al., 2011); members of the HD-Zip III subfamily mainly participate in different developmental events, such as cell differentiation, maintaining SAM and lateral organ polarity transport, and embryonic development (Prigge et al., 2005); members of the HD-Zip IV subfamily not only participate in plant root growth and development, anthocyanin accumulation, lipid biosynthesis and transport, but also regulate the expression of specific genes inside epidermal cells (Elhiti and Stasolla, 2009).

At present, the HD-Zip gene family has been identified and characterized in various plants. For example, 32 members of the HD-Zip gene family were identified in barley (*Hordeum vulgare* L.) (Li, 2020), 55 different members of the HD-Zip gene family were identified in corn (*Zea mays* L.) (Zhao, 2014), and 48 members were identified in *Arabidopsis thaliana* (Ariel et al., 2007). However, there is no relevant research on the oat HD-Zip gene family. This study is based on the Sanfensan genome of oats, using bioinformatics methods to identify members of the oat HD-Zip gene family, and analyze their protein physicochemical properties, chromosome location, gene structure, evolutionary relationship, and differential expression under drought and salt stress. This lays the foundation for the further study of the function and regulatory mechanism of the oat HD-Zip gene family and its application in the improvement of oat resistance.

## 2 Materials and methods

### 2.1 Acquisition of oat HD-Zip gene family sequences

The oat “Sanfensan” genome data and gff annotation files were downloaded from the NCBI website, and the HD-Zip protein sequence of *Arabidopsis thaliana* was downloaded from the Tair database as a reference sequence. Using TBtools (v2.011) (Chen et al., 2020) to obtain the “Sanfensan” protein sequence, while utilizing the online tool “Protein BLAST” from the National Center for Biotechnology Information (NCBI) to screen potential sequences of the HD-Zip gene family. Next, utilizing the online tool “Protein BLAST” from NCBI to screen potential sequences of the HD-Zip gene family. Based on the results of the HD-Zip sequence screening, the “CD-Search tool” in NCBI was used to obtain the conserved structural domains of the oat HD-Zip gene-encoded proteins, and the “Redraw Domain Pattern” in TBtools was used to visualize the conserved domains in oats.

### 2.2 Prediction of physicochemical properties of the oat HD-Zip gene family

The basic physicochemical properties of oat protein sequences, such as the isoelectric point and molecular weight, are predicted using the online website ExPasy (https://www.expasy.org/).

### 2.3 Analysis of oat gene structure and motifs

Using the annotation file of the oat genome, the specific locations of each HD-Zip gene on the chromosomes and the length of each chromosome are obtained. The “Visualize Gene Structure (Basic)” function in TBtools is used to visualize the gene structure of the selected sequences. The MEME tool (https://meme-suite.org/meme/doc/meme.html) (Bailey et al., 2009) is used to predict the conserved motifs of the oat HD-Zip gene family, with a maximum motif retrieval value to 10, selecting “Zero or One Occurrence Per Sequence” for motif distribution, and leave the remaining parameters as default values provided by MEME tool. For example, setting the motif discovery mode to Classic mode. The result file mast. xml is downloaded. The “Visualize MEME/MSAT Motif Pattern” in TBtools is used to visualize the conserved motifs.

### 2.4 Distribution of oat HD-Zip genes on chromosomes

Based on the annotation file of the oat genome, after obtaining information such as the corresponding starting positions on the chromosomes from the Sanfensan oat genome database, the “Gene Location Visualize from GTF/GFF” function in TBtools is used to create a map of the physical locations of the oat HD-Zip genes on the chromosomes.

### 2.5 Collinearity analysis of oat HD-Zip genes

Collinearity analysis of HD-Zip genes within the oat genome is performed using the “One Step MCScanX” function in TBtools, and a collinearity map within the genome is drawn using the Circos software.

### 2.6 Multiple sequence alignment of oat HD-Zip genes

The Clustal W program of the MEGA11.0 software (https://megasoftware.net/) is used to perform multiple sequence alignment of oat HD-Zip proteins, and the alignment results are loaded into the Jalview software (https://www.jalview.org/) for visualization of the multiple sequence alignment.

### 2.7 Construction of phylogenetic tree

The multiple sequence alignment results of the oat HD-Zip genes were used to construct a phylogenetic tree using the Maximum Likelihood (ML) method (with the bootstrap value set to 1,000, model set to Jones-Taylor-Thornton (JTT) + Gamma Distributed (G), partial deletion value set to 80%, and the rest of the parameters set to default). This was used to examines the evolutionary relationships of the oat HD-Zip family. The iTOL online tool (https://itol.embl.de/) is used for visualization and beautification of the results.

### 2.8 Analysis of cis-acting elements

Extract the promoter sequence of 2000 bp upstream of the starting site of the oat HD-Zip gene sequence. Use the PlantCARE online website (http://bioinformatics.psb.ugent.be/webtools/plantcare/html/) to predict the cis-acting elements of the promoter sequence. Visualize the prediction results using Tbtools.

### 2.9 Analysis of abiotic stress expression patterns of oat HD-Zip genes

Search for the original database number of the HD-Zip gene in the oat multi-stress transcriptome data (PRJNA727473) to obtain the FPKM (fragments per kilobase per million) values of the gene. Use the “HeatMap” function of TBtools to create a heatmap of gene expression, to explore the expression pattern of oat HD-Zip genes under abiotic stress.

### 2.10 HD-Zip transcription factor qRT-PCR validation

The three-leaf stage oat seedlings were subjected to PEG drought stress treatment, and the group without PEG treatment was taken as the control. The expression of HD Zip transcription factor genes was verified in oat seedlings and the control group seedlings treated with PEG for 12 h. The RNA were extracted from the samples using the TRIzol method, and then reverse transcribed into cDNA for quantitative PCR reactions, following the instructions of the Takara kit (RR820A) manual. The quantitative PCR primers for HD-Zip are designed using Primer Premier 5 software, as shown in the Table 1. The reference gene are chosen based on previous information, using the oat *ASUCH-4D* gene as the internal reference gene (Jia et al., 2022).

**TABLE 1:** Primer information.

Gene	Primer	Sequences (5′-3′)
*ASUCH-4D-2*	ASUCH-4D-2F	AGAGGACATGTGGTATTGCC
	ASUCH-4D-2R	GTTGGACATCTGCCTGCTTT
*Ashdz-35*	Ashdz-35F	CAGCACCCTAACCATGGAAC
	Ashdz-35R	TTGCAGCCTCCTGTTCTCCT
*Ashdz-37*	Ashdz-37F	CGCAGCTGGAAGAGCTGAAG
	Ashdz-37R	GGTTGTCGTACTCGTACCAG

## 3 Results

### 3.1 Identification and physicochemical property analysis of oat HD-Zip gene family members

The size of the oat “Sanfensan” genome is 10.0GB. In this study, 74 sequences of the HD-Zip gene family were identified in the oat genome. According to the Latin abbreviation of oats, the oat HD-Zip transcription factors were named *Ashdz1* through *Ashdz74*. After obtaining these 74 non-repetitive gene sequences, the relative molecular weight and isoelectric point of *Ashdzs* proteins were predicted using the Expasy website (Supplementary Table S1). The relative molecular weights range from 19,542.16 to 92,331.46 Da for *Ashdz31* to *Ashdz25*. Except for *Ashdz3* and *Ashdz13*, whose protein sequences have an isoelectric point greater than 10, the isoelectric points of the remaining protein sequences are between 4.47 and 9.95 (*Ashdz16* ∼ *Ashdz67*). The length of the oat HD-Zip protein is between 173 and 841 amino acids. Except for *Ashdz70*, *Ashdz58*, *Ashdz73*, *Ashdz68*, *Ashdz65*, *Ashdz47*, and *Ashdz25*, whose protein lengths are greater than 742 amino acids, the amino acid lengths of the remaining sequences are all within 173 ∼ 345 (*Ashdz31* ∼ *Ashdz57*). This shows that the oat HD-Zip genes and proteins have a large span in length.

### 3.2 Structural feature analysis of the oat HD-Zip gene family

**FIGURE 1:**
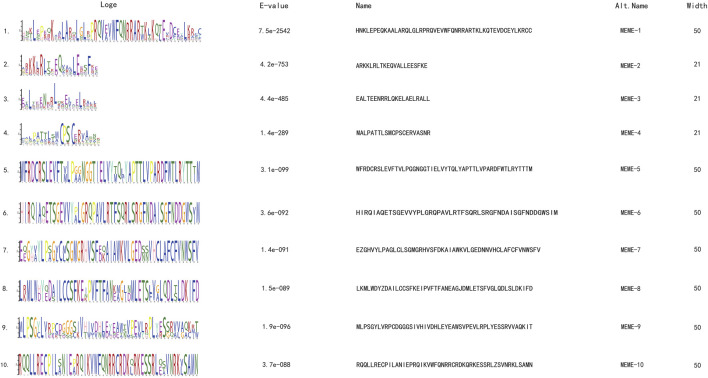
Oat HD-Zip gene motif identification.

**FIGURE 2:**
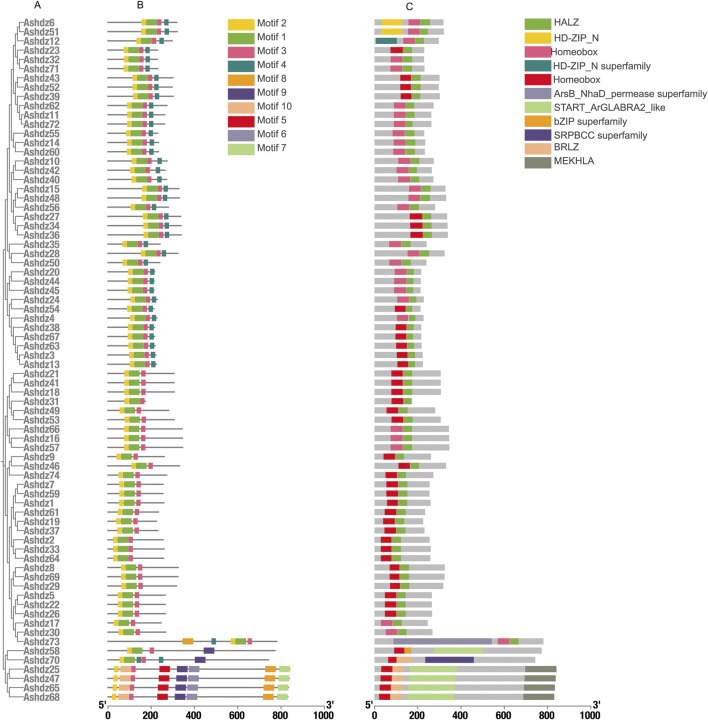
**(A)**Structural characterisation of the HD-Zip gene family in oats **(B)** Conserved motif analysis of the oat HD-Zip gene family; **(C)** Analysis of conserved structural domains of the oat HD-Zip gene family.

Motif analysis identified 10 conserved motifs across the 74 genes (Figure 1), and all proteins have motifs 1–3 (Figure 2B). Through the analysis of conserved domains (Figure 2C), it is known that Motif 1 and 2 constitute the HD homologous domain, and Motif 3 represents the LZ leucine zipper conserved sequence. Motifs 5, 6, and 9 form the START conserved domain, and Motif 7 and 8 constitute the MEKHLA conserved domain, which is a unique domain of the HD-Zip III subfamily (Sharif, 2021).

Based on the motif sequence analysis, the conserved protein domains of the four subfamilies HD-Zip Ⅰ, Ⅱ, Ⅲ, Ⅳ are screened. In the 74 gene family sequences, 30 of the HD-Zip Ⅰ subfamily, 38 of the HD-Zip Ⅱ subfamily, 4 of the HD-Zip Ⅲ subfamily, and 2 of the HD-Zip Ⅳ subfamily are selected respectively. By analyzing the structure distribution of motifs, it can be seen that the distribution of motifs within each subfamily is relatively consistent. Although the number of HD-Zip Ⅲ and HD-Zip Ⅳ subfamilies is the least, their motifs are richer than those of HD-Zip Ⅰ and HD-Zip Ⅱ.

### 3.3 Distribution of oat HD-Zip genes on chromosomes

**FIGURE 3:**
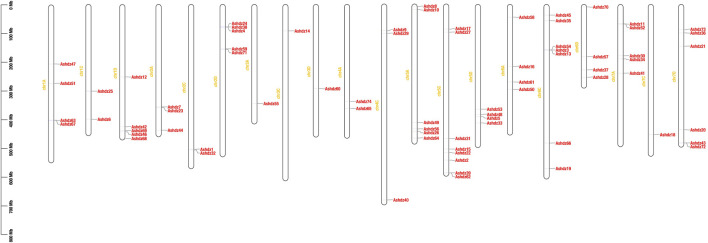
Distribution of HD-Zip genes on oat chromosomes.

Using the TBtools software to analyze the annotation file of the oat “Sanfensan” genome, the specific locations of 74 gene sequences on the chromosomes were obtained (Figure 3). The results show that the distribution of oat HD-Zip genes on each chromosome is not uniform. There is no distribution on the 4D chromosome, and the most distribution is on the 5C chromosome, with 8 genes; the least distribution is on the 3C, 3D, and 7C chromosomes, each with only 1 gene. At the same time, the distribution of HD-Zip genes on each chromosome is also uneven. In some chromosomes, there are regions where gene distribution is relatively concentrated, such as the end of the 1D chromosome, the end of the 5C chromosome, and the upper end of the 6C chromosome. Gene clusters were also found on the 1A, 2D, 5C, 6C, 7A, and 7D chromosomes.

### 3.4 Collinearity analysis of oat HD-Zip genes

**FIGURE 4:**
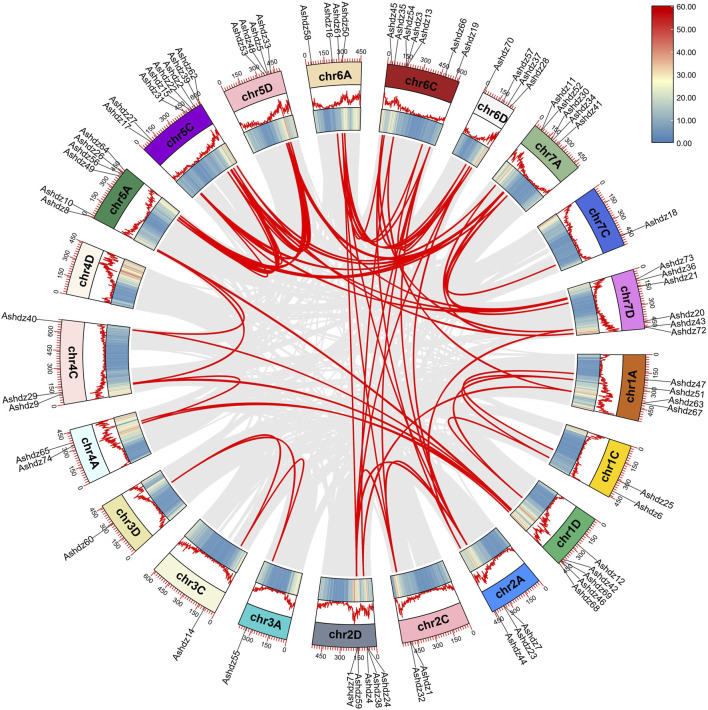
Collinearity relationships of the oat HD-Zip gene family members.

Through genome-wide collinearity analysis of the oat HD-Zip gene (Figure 4), a significant number of chromosomal segment duplication events (Segmental duplication) were identified within the HD-Zip gene family. Chromosomal segment duplication is a fundamental process in the evolution of gene families, resulting from chromosomal recombination, and is typically considered the primary driver for the expansion of gene families within the genome. As depicted in the figure, apart from the 4D chromosome which lacks HD-Zip genes and therefore does not exhibit chromosomal duplication events, all other oat chromosomes have experienced chromosomal duplication events. However, the distribution of genes across the chromosomes is not even, with the 7C chromosome having the fewest events, only one. The 74 genes within the family have given rise to 84 pairs of chromosomal duplication events, suggesting that more than one gene has undergone multiple chromosomal duplication events. *Ashdz7* has the highest occurrence, appearing 5 times.

### 3.5 Phylogenetic analysis of oat HD-Zip genes

**FIGURE 5:**
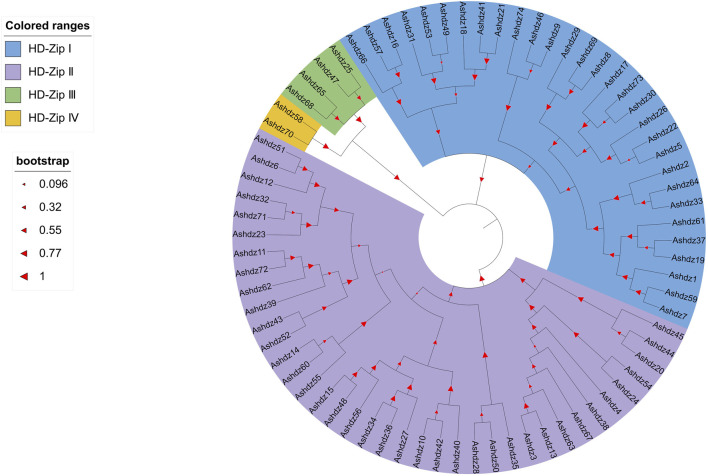
Phylogenetic tree of oat HD-Zip transcription factors.

**FIGURE 6:**
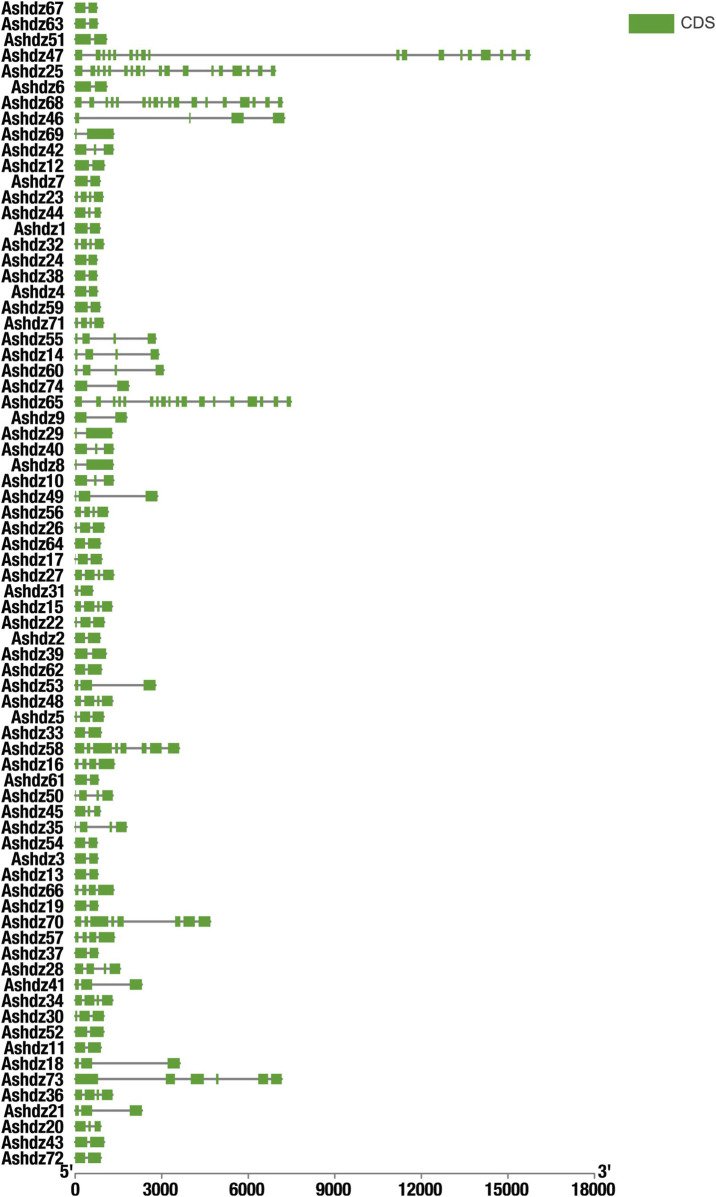
Analysis of HD-Zip Gene Structure Green represents the CDS coding region; solid lines represent the intron non-coding region.

After blasting and screening the entire oat genome, 74 HD-Zip transcription factor sequences were obtained. These sequences all have high homology and conservation. Therefore, in order to analyze the phylogenetic relationship of HD-Zip transcription factors in oats, the MEGA 11 software was used to construct the phylogenetic tree of the oat HD-Zip gene family using the Maximum Likelihood (ML) method (Figure 5). The results showed that the phylogenetic tree divided the 74 protein sequences into four branches (HD-Zip Ⅰ, Ⅱ, Ⅲ, Ⅳ), which is consistent with the results of the conservative base sequence analysis. Subfamily Ⅰ and subfamily Ⅱ have the largest proportion, while subfamily Ⅲ and Ⅳ have the smallest proportion. In the evolutionary relationship, it can be seen that subfamily Ⅲ and subfamily Ⅳ first cluster into a branch on the phylogenetic tree and then aggregate with subfamily Ⅰ and Ⅱ, which proves that HD-Zip Ⅲ and HD-Zip Ⅳ have a close phylogenetic relationship. A total of 25 pairs of genes appeared in pairs, and apart from the 7 pairs of genes *Ashdz32/71*, *Ashdz14/60*, *Ashdz10/42*, *Ashdz28/50*, *Ashdz49/53*, *Ashdz9/46*, *Ashdz5/22*, the remaining gene pairs all have high the bootstrap values. In order to further support the HD-Zip phylogenetic tree, the gene structure of 74 HD-Zip transcription factors was compared and analyzed (Figure 6). From the figure, it can be seen that the gene structure of oat HD-Zip is quite special, unlike the HD-Zip gene structure of crops such as corn and Arabidopsis (Zhao, 2014; Ariel et al., 2007), which includes three parts: introns, exons, and UTR regions. The gene structure of the entire HD-Zip transcription factor in oats only includes two parts, namely introns and exons, which may be due to the difference in gff annotation files. Each coding sequence of the HD-Zip gene is divided into several sections by one or more introns, and from the figure, it can be known that subfamily HD-Zip Ⅲ contains the most introns, and HD-Zip I and II subfamilies have the least number of introns.

### 3.6 Analysis of cis-acting elements in the promoter of oat HD-Zip genes

**FIGURE 7:**
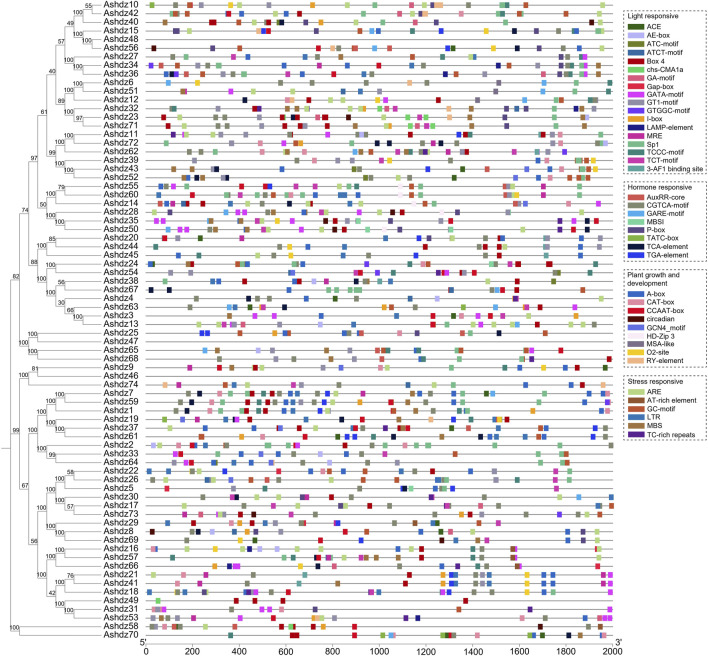
Analysis of cis-acting elements in the 2000 bp upstream promoter of oat HD-Zip genes.

Previous studies have shown that the HD-Zip gene family is involved in responding to abiotic stress in crops (Kang, 2014). By analyzing the prediction results of the oat HD-Zip gene sequence on the online website PlantCARE (Figure 7), we selected cis-acting elements related to stress response and hormone response for analysis. The results showed that a total of 1,324 cis-acting elements were identified in 74 oat HD-Zip sequences, of which the most common being those related to light response, totaling 527, and the cis-acting elements related to growth and development appeared the least, totaling 235. Moreover, the cis-acting elements related to light response are also the most widely distributed type. Except for not being found in *Ashdz48*, *Ashdz47*, *Ashdz46* these three genes, they appear in the remaining 71 genes. The cis-acting elements related to hormones, growth and development, and stress are less distributed, only appearing in 68 genes. Therefore, it can be known that the oat HD-Zip gene may be related to the above biological functions and plays an important role in light response, hormones and abiotic stress, and regulating the growth and development process of oats.

### 3.7 Analysis of the abiotic stress expression pattern of oat HD-Zip genes

#### 3.7.1 Transcriptome expression profile of oat HD-Zip genes under drought stress conditions

**FIGURE 8:**
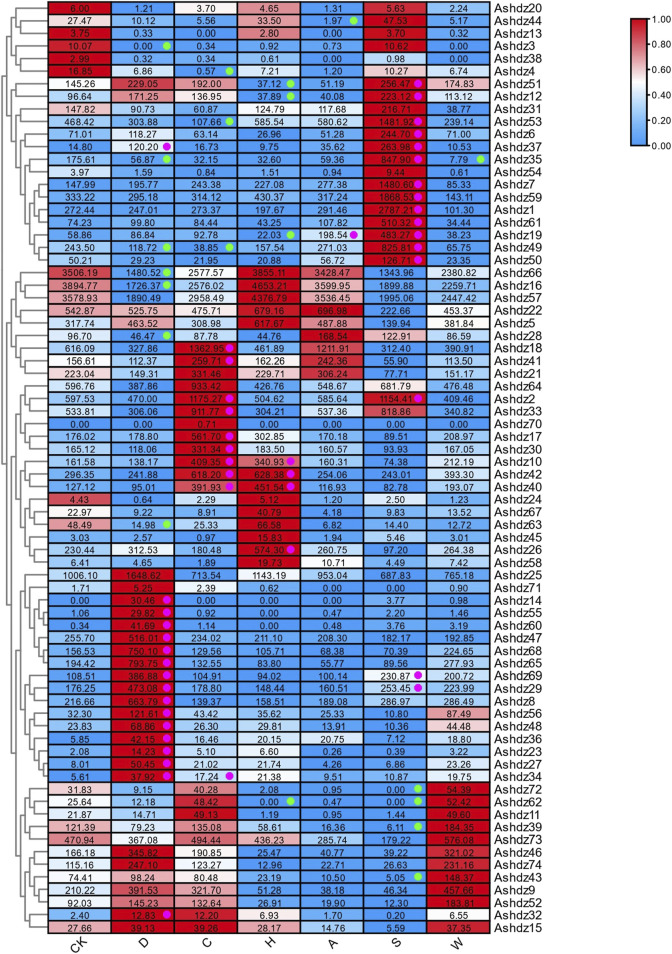
Expression profile of oat HD-Zip genes in response to single stress CK: Control group; D: Drought stress; C: Cold stress; H: Heat stress; A: Alkali stress; S: Salt stress; W: Waterlogging stress; Green dots represent significantly downregulated expression; pink dots represent significantly upregulated expression.

Based on the public RNA-seq data under drought stress, the expression profile of oat HD-Zip genes was analyzed (Figure 8). In general, a total of 24 HD-Zip genes were significantly induced or suppressed. *Ashdz8*, *Ashdz49*, *Ashdz66*, *Ashdz16* showed higher expression levels under both control and drought stress conditions. Among these 24 genes, the expression levels of 17 genes increased due to drought stress response, while the expression levels of the other 7 genes decreased. Among these, *Ashdz63*, *Ashdz3*, *Ashdz35*, and *Ashdz28* displayed lower expression levels under both control and stress conditions; notably, the expression of the *Ashdz3* gene was completely suppressed under drought stress, dropping to 0. The expression of *Ashdz66* and *Ashdz16* was also inhibited under drought stress, with their expression levels dropping to about 50% of the control group.

Among the 17 HD-Zip genes with increased expression, the expression of the Ashdz14 gene is particularly unique. Its expression level is 0 under normal conditions, but when subjected to drought stress, the expression of this gene is upregulated by 24 ∼ 36 times. The expression level of *Ashdz65* gene is the highest under drought conditions.

#### 3.7.2 Transcriptome expression profile of oat HD-Zip genes under multiple stress conditions

In the salt stress RNA-seq data, 20 oat HD-Zip genes were either induced or repressed (Figure 8). The results show that among these 20 genes, the expression level of 16 genes is upregulated in response to salt stress. Among the upregulated genes, *Ashdz1* has the highest expression level, and *Ashdz37* and *Ashdz19* show a strong trend of induced upregulation, which are on average 26 times and 11 times higher than the control group, respectively. The expression level of 4 genes is downregulated, among which the expression levels of *Ashdz62* and *Ashdz72* drop to 0 under salt stress.

Among the genes that are significantly upregulated or downregulated in drought and salt stress, *Ashdz29*, *Ashdz49*, *Ashdz37*, *Ashdz69*, *Ashdz35* these five genes are expressed in both. However, *Ashdz49*, *Ashdz35* are downregulated under drought stress, while *Ashdz29*, *Ashdz37*, *Ashdz69* are upregulated in response to drought stress and salt stress. Compared to the control, *Ashdz37* shows a strong trend of induced upregulation in both stresses, upregulating on average 8 times in drought stress and 26 times in salt stress. It can be speculated that the *Ashdz37* gene plays an important role in the regulation of abiotic stress in oats.

### 3.8 qRT-PCR validation of differentially expressed HD-Zip genes

**FIGURE 9:**
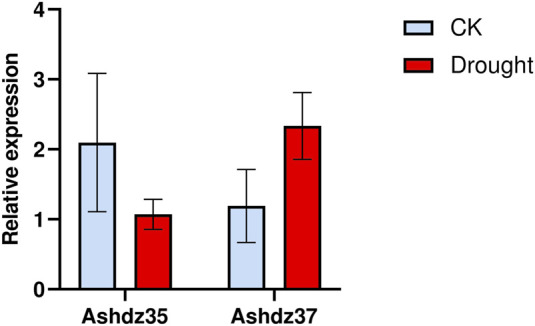
Comparison of qRT-PCR gene expression levels of *Ashdz35* and *Ashdz37*.

To verify the accuracy of the oat transcriptome data, based on the HD-Zip differential gene expression profile, two genes with significant differential expression, *Ashdz35* and *Ashdz37*, were selected. The relative expression levels of these two genes under normal conditions and drought stress were measured using quantitative PCR, and the results are shown in Figure 9. From Figure 9, it can be seen that the gene *Ashdz35* has a high relative expression level under normal conditions and its expression level decreases under drought stress. The *Ashdz37* gene has a low relative expression level under normal conditions, but its expression level significantly increases under drought stress. This is consistent with the trend in the transcriptome data, which proves the accuracy of the transcriptome data.

## 4 Discussion

Plant transcription factors often regulate the growth and development process of plants by specifically binding to DNA and other proteins or transcription factors to promote or inhibit gene expression (Deng, 2014). An increasing number of research findings indicate that HD-Zip transcription factors are involved in plant responses to biotic and abiotic stresses. In Arabidopsis, the involvement of three HD-Zip transcription factors, *ATHB6*, *ATHB7*, and *ATHB12*, in drought stress responses has been validated (Aoyama et al., 1995). The rice HD-Zip gene *Oshox22* has been shown to participate in the synthesis of abscisic acid (ABA) and regulate plant responses to drought and salt stress through the ABA signaling pathway (Zhang et al., 2012). In *Craterostigma plantagineum*, the expression of two HD-Zip genes, *CpHB6* and *CpHB7*, has been found to be induced by drought and ABA (Deng et al., 2002). These research findings indicate that HD-Zip transcription factors play a crucial role in regulating plant responses to various abiotic stress factors. However, studies on transcription factors in oats are still limited.

This study systematically researched the oat HD-Zip gene family through bioinformatics methods. A total of 74 HD-Zip gene family members were identified from the whole genome data of oat Sanfensan, unevenly distributed on 21 chromosomes. According to the gene structure, conserved domains, and evolutionary relationships, these sequences can be further divided into four subfamilies HD-Zip Ⅰ-Ⅳ, which aligns with the classifications observed in barley, corn, and Arabidopsis. that the HD-Zip subfamily in oats exhibits structural similarity. HD-Zip Ⅰ only contains HD domain and LZ motif; HD-Zip Ⅱ, in addition to HD and LZ conserved domains, also has a highly conserved N-terminus (Shao et al., 2020); HD-Zip Ⅲ not only has HD and LZ conserved domains, but also has a lipid transport domain (START) and MEKHLA motif; the structure of HD-Zip Ⅳ is missing the MEKHLA motif compared to HD-Zip Ⅲ (Guo et al., 2021). Through the analysis of gene collinearity within the species, a total of 85 chromosome segment duplication events were identified. Chromosome segment duplications involving *Ashdzs* were found on all chromosomes except 4D, indicating that segment duplication may be the main reason for the expansion of the *Ashdzs* gene family. The uneven distribution of HD-Zip genes on chromosomes has also been reported in other species. For example, in wheat, there is no distribution of HD-Zip genes on chromosomes 7B, 6B, and 3A (Huang et al., 2018). No HD-Zip gene distribution was detected on chromosome Chr08 in watermelon (Yan et al., 2022). According to the results of the phylogenetic tree of oat HD-Zip genes, the kinship between HD-Zip Ⅲ and HD-Zip Ⅳ is closer, which is consistent with the results of conserved motif and gene structure analysis. It can be inferred that the two major branches of subfamily Ⅰ and Ⅱ and subfamily Ⅲ and Ⅳ come from different ancestors, and the structure of HD-Zip Ⅲ and Ⅳ is more complex compared to HD-Zip Ⅰ and Ⅱ, which indicates that HD-Zip Ⅲ and Ⅳ may have a higher degree of evolution.

The RNA-seq data of oat plants under drought and salt stress revealed the HD-Zip genes that were significantly upregulated or downregulated induced by these stresses. The five genes *Ashdz29*, *Ashdz49*, *Ashdz37*, *Ashdz69*, *Ashdz35* were all significantly induced or suppressed under drought and salt stress. Combined with the analysis of cis-acting elements, it is known that except for *Ashdz49* which only contains light-responsive cis-elements, the other four genes all have four types of cis-elements. In particular, *Ashdz37* shows a strong trend of induced upregulation in the RNA-seq data under both stresses, and it also has four types of cis-elements related to light response, hormones and abiotic stress, and growth and development. By analyzing the types of cis-elements in *Ashdz37*, it was found that this gene not only contains the MBS cis-element, which is directly involved in drought-induced responses, but also includes cis-acting elements such as the CCAAT-box, AuxRR-core, and CGTCA-motif, which could indirectly influence responses to drought and salt stress, such as seed development, jasmonic acid response, and auxin response. Therefore, it can be speculated that *Ashdz37* can not only directly participate in drought-induced and salt stress responses, but also indirectly participate in the stress resistance response of oats by affecting physiological regulation such as auxin response and seed development. It is a key regulatory gene in oats responding to drought and salt stress.

## 5 Conclusion

Using bioinformatics methods, 74 HD-Zip gene sequences were identified from the whole genome of oats, and they were named in sequence as *Ashdz1*-*74*. The conserved domains, conserved motifs, and phylogenetic relationships of these sequences were analyzed. On this basis, the 74 gene sequences were divided into four subfamilies (HD-Zip Ⅰ-Ⅳ). Through the analysis of expression patterns, it is speculated that the gene *Ashdz37* may play an important role in the regulation process of oat stress response. These findings provide a theoretical foundation for future functional research and characterization of the HD-Zip gene family in oats.

## Data Availability

The datasets presented in this study can be found in online repositories. The names of the repository/repositories and accession number(s) can be found in the article/Supplementary Material.
