# Supplementary material for: Genome-wide identification and analysis of the HD-Zip transcription factor family in oats
Source: Front Mol Biosci. 2024 Oct 30;11:1475276. doi: 10.3389/fmolb.2024.1475276 (PMC11557432; doi:10.3389/fmolb.2024.1475276)
Supplement: Supplementary file 1 [file Table1.docx]

Annexed table.1 Physicochemical properties of the oat HD-zip gene family protein sequences

| Gene ID | Original database ID | Chr | Location | | Protein length(aa) | Isoelectric point | Molecular weight(Da) |
| --- | --- | --- | --- | --- | --- | --- | --- |
| Ashdz1 | A.satnudSFS2C01G003681.1 | chr2C | 504331813-504332671 | 258 | | 5.21 | 28873.02 |
| Ashdz2 | A.satnudSFS5C01G004297.1 | chr5C | 543304026-543304893 | 254 | | 5.15 | 27821.84 |
| Ashdz3 | A.satnudSFS6C01G003184.1 | chr6C | 158737006-158737791 | 222 | | 10.01 | 24359.48 |
| Ashdz4 | A.satnudSFS2D01G001590.1 | chr2D | 78906912-78907682 | 226 | | 9.07 | 24830.91 |
| Ashdz5 | A.satnudSFS5D01G004031.1 | chr5D | 390846490-390847481 | 264 | | 4.76 | 28724.83 |
| Ashdz6 | A.satnudSFS1C01G002599.1 | chr1C | 399622571-399623664 | 318 | | 9.03 | 34403.51 |
| Ashdz7 | A.satnudSFS2A01G002869.1 | chr2A | 356751289-356752148 | 254 | | 5.31 | 28527.75 |
| Ashdz8 | A.satnudSFS5A01G000146.1 | chr5A | 8509378-8510693 | 324 | | 5.13 | 35521.28 |
| Ashdz9 | A.satnudSFS4C01G002335.1 | chr4C | 92802343-92804127 | 260 | | 5.61 | 28244.42 |
| Ashdz10 | A.satnudSFS5A01G000473.1 | chr5A | 20093642-20094970 | 273 | | 7.6 | 29412.86 |
| Ashdz11 | A.satnudSFS7A01G003970.1 | chr7A | 67948930-67949819 | 262 | | 8.9 | 27516.1 |
| Ashdz12 | A.satnudSFS1D01G005175.1 | chr1D | 251981787-251982798 | 296 | | 9.41 | 31952.85 |
| Ashdz13 | A.satnudSFS6C01G003185.1 | chr6C | 158745524-158746312 | 223 | | 10.06 | 24261.41 |
| Ashdz14 | A.satnudSFS3C01G001507.1 | chr3C | 92371837-92374732 | 234 | | 9.39 | 26069.55 |
| Ashdz15 | A.satnudSFS5C01G003540.1 | chr5C | 504880867-504882146 | 327 | | 7.64 | 34314.14 |
| Ashdz16 | A.satnudSFS6A01G001632.1 | chr6A | 216319427-216320782 | 344 | | 4.73 | 37418.06 |
| Ashdz17 | A.satnudSFS5C01G000760.1 | chr5C | 87492070-87492990 | 245 | | 4.79 | 26823.68 |
| Ashdz18 | A.satnudSFS7C01G004103.1 | chr7C | 453202335-453205960 | 306 | | 4.94 | 33642.01 |
| Ashdz19 | A.satnudSFS6C01G005581.1 | chr6C | 570917712-570918501 | 224 | | 4.78 | 25353.87 |
| Ashdz20 | A.satnudSFS7D01G004122.1 | chr7D | 436411126-436412010 | 215 | | 9.21 | 23769.67 |
| Ashdz21 | A.satnudSFS7D01G001943.1 | chr7D | 145383172 145385484 | 305 | | 4.95 | 33448.75 |
| Ashdz22 | A.satnudSFS5C01G003718.1 | chr5C | 516660776-516661784 | 264 | | 4.73 | 28772.84 |
| Ashdz23 | A.satnudSFS2A01G002920.1 | chr2A | 358829940-358830901 | 229 | | 8.92 | 25417.79 |
| Ashdz24 | A.satnudSFS2D01G001587.1 | chr2D | 78696605-78697362 | 227 | | 9.82 | 24714.77 |
| Ashdz25 | A.satnudSFS1C01G001900.1 | chr1C | 301757653-301764585 | 841 | | 5.54 | 92331.46 |
| Ashdz26 | A.satnudSFS5A01G004689.1 | chr5A | 443228753-443229756 | 265 | | 4.73 | 28847.92 |
| Ashdz27 | A.satnudSFS5C01G000839.1 | chr5C | 97973063-97974395 | 335 | | 6.28 | 35373.49 |
| Ashdz28 | A.satnudSFS6D01G002282.1 | chr6D | 253326348-253327905 | 323 | | 7.04 | 35403.8 |
| Ashdz29 | A.satnudSFS4C01G002482.1 | chr4C | 101967909-101969188 | 317 | | 5.24 | 34769.54 |
| Ashdz30 | A.satnudSFS7A01G002005.1 | chr7A | 178685513-178686504 | 267 | | 4.86 | 28972.04 |
| Ashdz31 | A.satnudSFS5C01G003140.1 | chr5C | 467711460-467712067 | 173 | | 7.03 | 19542.16 |
| Ashdz32 | A.satnudSFS2C01G003728.1 | chr2C | 506726654-506727641 | 228 | | 9.08 | 25386.75 |
| Ashdz33 | A.satnudSFS5D01G004636.1 | chr5D | 412485892-412486791 | 259 | | 5.14 | 28235.23 |
| Ashdz34 | A.satnudSFS7A01G001915.1 | chr7A | 189770585-189771881 | 337 | | 6.2 | 35678.82 |
| Ashdz35 | A.satnudSFS6C01G001194.1 | chr6C | 56382910-56384691 | 240 | | 9.47 | 26893.36 |
| Ashdz36 | A.satnudSFS7D01G001694.1 | chr7D | 99314588-99315881 | 338 | | 6.49 | 35703.92 |
| Ashdz37 | A.satnudSFS6D01G001716.1 | chr6D | 227571176-227571969 | 230 | | 4.97 | 25969.65 |
| Ashdz38 | A.satnudSFS2D01G001589.1 | chr2D | 78803650-78804407 | 215 | | 9.82 | 23351.38 |
| Ashdz39 | A.satnudSFS5C01G005396.1 | chr5C | 587809156-587810225 | 301 | | 9.58 | 31965.57 |
| Ashdz40 | A.satnudSFS4C01G006007.1 | chr4C | 681794510-681795833 | 271 | | 7.62 | 29081.51 |
| Ashdz41 | A.satnudSFS7A01G001652.1 | chr7A | 239299633-239301944 | 305 | | 4.95 | 33448.75 |
| Ashdz42 | A.satnudSFS1D01G001456.1 | chr1D | 425363139-425364458 | 264 | | 7.62 | 28405.82 |
| Ashdz43 | A.satnudSFS7D01G005429.1 | chr7D | 482014191-482015195 | 300 | | 9.58 | 31794.42 |
| Ashdz44 | A.satnudSFS2A01G004772.1 | chr2A | 437775390-437776268 | 213 | | 9.32 | 23937.95 |
| Ashdz45 | A.satnudSFS6C01G000888.1 | chr6C | 37184058-37184925 | 212 | | 9.02 | 23895.93 |
| Ashdz46 | A.satnudSFS1D01G000972.1 | chr1D | 443118336-443125588 | 330 | | 6.02 | 35395.43 |
| Ashdz47 | A.satnudSFS1A01G006052.1 | chr1A | 205356959-205372716 | 838 | | 5.43 | 92021.95 |
| Ashdz48 | A.satnudSFS5D01G003851.1 | chr5D | 382832104-382833400 | 330 | | 6.67 | 34532.29 |
| Ashdz49 | A.satnudSFS5A01G004173.1 | chr5A | 412178435-412181285 | 280 | | 5 | 30856.14 |
| Ashdz50 | A.satnudSFS6A01G002719.1 | chr6A | 295433660-295434961 | 239 | | 9.59 | 26740.04 |
| Ashdz51 | A.satnudSFS1A01G005418.1 | chr1A | 272569427-272570510 | 320 | | 9.39 | 34503.72 |
| Ashdz52 | A.satnudSFS7A01G003969.1 | chr7A | 68071423-68072410 | 296 | | 9.58 | 31248.87 |
| Ashdz53 | A.satnudSFS5D01G003485.1 | chr5D | 364512335-364515122 | 305 | | 5.11 | 33155.78 |
| Ashdz54 | A.satnudSFS6C01G003183.1 | chr6C | 158680716-158681477 | 212 | | 9.82 | 23161.03 |
| Ashdz55 | A.satnudSFS3A01G002979.1 | chr3A | 344899912-344902704 | 229 | | 9.27 | 25551.06 |
| Ashdz56 | A.satnudSFS5A01G004535.1 | chr5A | 434536987-434538124 | 279 | | 9.41 | 29941.71 |
| Ashdz57 | A.satnudSFS6D01G001158.1 | chr6D | 181971302-181972660 | 345 | | 4.77 | 37563.26 |
| Ashdz58 | A.satnudSFS6A01G000655.1 | chr6A | 44546170-44549773 | 772 | | 5.99 | 83690.53 |
| Ashdz59 | A.satnudSFS2D01G003004.1 | chr2D | 154615062-154615928 | 253 | | 5.21 | 28438.63 |
| Ashdz60 | A.satnudSFS3D01G001994.1 | chr3D | 293157190-293160259 | 232 | | 9.27 | 25893.38 |
| Ashdz61 | A.satnudSFS6A01G002197.1 | chr6A | 270244128-270244933 | 233 | | 4.97 | 26209.91 |
| Ashdz62 | A.satnudSFS5C01G005397.1 | chr5C | 587884533-587885445 | 272 | | 8.84 | 28496.06 |
| Ashdz63 | A.satnudSFS1A01G002885.1 | chr1A | 401386407-401387184 | 217 | | 9.27 | 23768.59 |
| Ashdz64 | A.satnudSFS5A01G005301.1 | chr5A | 466585646-466586522 | 257 | | 5.15 | 28080.07 |
| Ashdz65 | A.satnudSFS4A01G005071.1 | chr4A | 361957676-361965155 | 834 | | 5.9 | 91120.75 |
| Ashdz66 | A.satnudSFS6C01G004994.1 | chr6C | 482636033-482637367 | 343 | | 4.75 | 37270.97 |
| Ashdz67 | A.satnudSFS1A01G002884.1 | chr1A | 401418730-401419486 | 215 | | 9.95 | 23405.55 |
| Ashdz68 | A.satnudSFS1D01G000262.1 | chr1D | 465976898-465984076 | 831 | | 5.97 | 90952.62 |
| Ashdz69 | A.satnudSFS1D01G001104.1 | chr1D | 437928127-437929459 | 323 | | 5.13 | 35500.22 |
| Ashdz70 | A.satnudSFS6D01G000115.1 | chr6D | 8877424-8882107 | 742 | | 7.28 | 80762.37 |
| Ashdz71 | A.satnudSFS2D01G003058.1 | chr2D | 156518436-156519415 | 230 | | 8.92 | 25559.95 |
| Ashdz72 | A.satnudSFS7D01G005431.1 | chr7D | 482046573-482047464 | 262 | | 8.9 | 27481.16 |
| Ashdz73 | A.satnudSFS7D01G001593.1 | chr7D | 88180838-88187998 | 780 | | 6.46 | 83645.54 |
| Ashdz74 | A.satnudSFS4A01G004361.1 | chr4A | 337608205-337610063 | 271 | | 5.87 | 29172.48 |
